# Long Noncoding RNAs in Imprinting and X Chromosome Inactivation

**DOI:** 10.3390/biom4010076

**Published:** 2014-01-06

**Authors:** Joseph M. Autuoro, Stephan P. Pirnie, Gordon G. Carmichael

**Affiliations:** Department of Genetics and Developmental Biology, University of Connecticut Health Center, 400 Farmington Avenue, Farmington, CT 06030, USA; E-Mails: jautuoro@gmail.com (J.M.A.); spirnie@student.uchc.edu (S.P.P.)

**Keywords:** imprinting, long noncoding RNA, XIST, KCNQOT1, Igf2r, Airn, SNURF-SNRPN, UBE3A, DLK1-DIO3, H19-IGF2

## Abstract

The field of long noncoding RNA (lncRNA) research has been rapidly advancing in recent years. Technological advancements and deep-sequencing of the transcriptome have facilitated the identification of numerous new lncRNAs, many with unusual properties, however, the function of most of these molecules is still largely unknown. Some evidence suggests that several of these lncRNAs may regulate their own transcription in cis, and that of nearby genes, by recruiting remodeling factors to local chromatin. Notably, lncRNAs are known to exist at many imprinted gene clusters. Genomic imprinting is a complex and highly regulated process resulting in the monoallelic silencing of certain genes, based on the parent-of-origin of the allele. It is thought that lncRNAs may regulate many imprinted loci, however, the mechanism by which they exert such influence is poorly understood. This review will discuss what is known about the lncRNAs of major imprinted loci, and the roles they play in the regulation of imprinting.

## 1. Introduction

Genomic imprinting is an epigenetic phenomenon, whereby differential expression of alleles occurs with respect to the parent-of-origin. It is thought to have evolved in placental mammals as a biological/ecological mechanism for dosage compensation, whereby a conflict of interest exists over maternal investment of resources during gestation, and, thus, dosage mediation (particularly of growth-related genes) [1,2,3]. The existence of imprinted genes was hypothesized after experiments demonstrated the failure of parthenogenetic embryos to develop, as well as failure of embryos created from bi-maternal or bi-paternal pronuclei [4,5], unless certain imprinted genes were deleted [6,7,8,9,10]. Imprinting events occur on most mammalian chromosomes, but only about 100 genes (less than 1% of known genes) are thought to be imprinted [1,11].

Imprinting is a process by which epigenetic marks are laid down at specific loci, based on the sex of the parent of origin of the chromosome, and usually leads to expression of genes from only one chromosome. Imprinting is characterized primarily by DNA methylation marks on special CpG-rich regulatory elements called Imprinting Control Regions (ICRs), catalyzed by the *de novo* DNA methyltransferases DNMT3A and DNMT3L, and later propagated by the maintenance DNA methyltransferase DNMT1 [12,13,14], and the ZFP57/KAP1 complex, which binds to a TGCCGC consensus motif on ICRs [15]. It is important to note that a Differentially Methylated Region (DMR) is not the same as an Imprinting Control Region (ICR), although they are both differentially methylated regions. The ICR is considered the governing region, whose methylation marks are by definition laid down *in the germline*, whereas somatic DMRs leading to imprinting are methylated in post-implantation embryos, and are considered secondary in the regulation hierarchy [12,16]. It is also important to note that the term imprinted does not specifically refer to gene expression status, but technically the *methylation* status of the ICR. In general, though, a methylated ICR usually correlates with a subsequently silenced allele [11,17].

**Figure 1:**
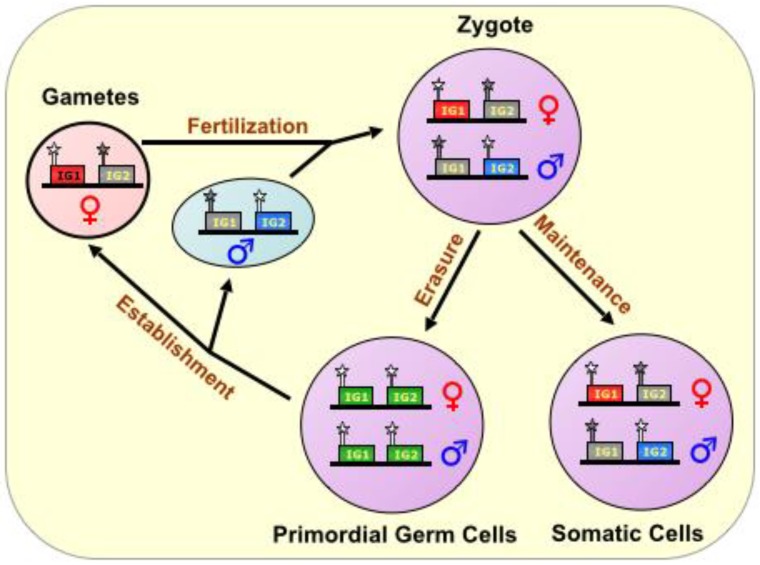
The Imprinting Cycle. Imprints are established during gametogenesis in a sex-specific manner, and are characterized primarily by DNA methylation marks on special CpG-rich regulatory elements called Imprinting Control Regions (ICRs), During fertilization, the complementation of haploid genomes, each with an epigenetic signature identifying the sex of the parent, produces a diploid offspring. In all future somatic tissue lineages, the imprinting marks are maintained, but in the nascent primordial germ cells, the parental imprinting marks are erased, and their germline descendants re-establish imprinting marks according to the sex of the individual, thus perpetuating the cycle. ICR is depicted as a star lollipop; white is unmethylated, gray is methylated. Red boxes indicate maternally expressed genes, blue boxes indicate paternally expressed genes, green boxes indicate biallelically-expressed genes, and gray boxes indicate silenced genes. “IG” stands for “imprinted gene.”

During fertilization, the complementation of haploid genomes, each with an epigenetic signature identifying the sex of the parent, produces a diploid offspring. Thereafter, genome-wide erasure of existing epigenetic modifications, on both DNA and histones, occurs throughout the pre-implantation embryo, but the imprinting marks persist via poorly understood mechanisms. In all future somatic tissue lineages, the imprinting marks are later followed by further epigenetic changes during development, resulting in a subset of genes being expressed monoallelically, from either the maternal or paternal chromosome. However, in the nascent primordial germ cells, the remaining parental imprinting marks are indeed erased, and their germline descendants re-establish imprinting marks according to the sex of the individual, thus perpetuating the cycle [13,14]. (See also Figure 1).

Interestingly, most imprinted genes are found in clusters [1,14]. These clusters usually feature a complex balance of both maternally- and paternally-imprinted genes in the same (often megabase-sized) locus, and many of the clusters are regulated by (and regulate) the transcriptional activity of a long noncoding RNA (lncRNA) [12]. Specifically, active transcription of the cluster’s lncRNA is linked to the reciprocal silencing of the other (mostly protein-coding) genes in the locus [11,16,18]. It is thought that these lncRNAs act *in cis*, by recruiting chromatin remodeling factors and other enzymes, which act *in trans*, to cooperatively regulate gene expression [11,19].

Some imprinted genes also bear tissue-specific and/or temporally-regulated imprinted gene expression, suggesting the involvement of additional factors in executing the functional status of those genes. Indeed, many imprinted genes are known to be involved in growth and development, particularly during pre- and peri-natal life [3,11,20,21]. In addition, a significant proportion of imprinted genes are expressed predominantly in the brain, suggesting an important role in higher-order brain processes, such as learning and behavior [2,22,23]. Perturbations of imprinted loci can result in pathological manifestations including Prader-Willi Syndrome, Angelman Syndrome, Beckwith-Wiedemann Syndrome, and Silver-Russell Syndrome [3,24], as well as some cancers [25].

This review will address what is known about the mechanisms of imprinting in six of the most well-studied imprinted gene clusters: *Kcnq1/Kcnq1ot1*, *SNURF-SNRPN/UBE3A*, *DLK1-DIO3/MEG3*, *H19/IGF2*, *Igf2r/Airn*, as well as X chromosome inactivation. Not included in this review are some other known imprinted lncRNAs, such as *GNAS* and *HOTAIR*, which are reviewed in several of the sources cited herein. In addition, we do not wish to disregard the importance of *small* noncoding RNAs (i.e., snoRNAs, miRNAs, piRNAs, *etc*.) in these imprinted loci [26], but for the purposes of this review, we will focus primarily on imprinted *long* noncoding RNAs.

## 2. XIST and X-Chromosome Inactivation

In mammals, the XY sex-determination system bestows females with two X chromosomes, and males with one X and one Y, thus necessitating a dosage equalization mechanism for most X-linked genes. X chromosome inactivation (XCI) occurs stochastically in female post-implantation embryonic somatic cells—that is, either the maternal or paternal X chromosome is randomly silenced in every non-germline cell of the embryo proper. Once established, the same inactive X chromosome is consistently maintained in all future daughter cells.

**Figure 2:**
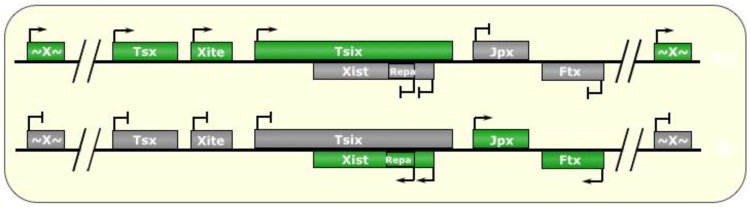
X-Chromosome Inactivation. In post-implantation female mammalian cells, one of the two X chromosomes is randomly silenced, bearing a chromatin signature that is passed down to all future daughter cells. The X inactivation center (XIC) is host to several noncoding RNAs that regulate this process. At around the implantation stage of early embryogenesis, both chromosomes are active, and both express the *Tsix* lncRNA, which negatively regulates its own antisense transcript, *Xist*. An incompletely understood molecular counting mechanism then fosters the choice of one *Tsix* allele on the future active chromosome (Xa) to continue being expressed, and the other allele on the future inactive X chromosome (Xi) is silenced. As a result, *Xist* is de-repressed on the Xi, and the RNA begins to accumulate and spreads to form an RNA cloud *in cis* all around the future inactive X chromosome (Xi). This is accompanied by recruitment of heterochromatic remodeling enzymes, such as PRC2, which writes H3K27me3 marks chromosome-wide, to silence the majority of genes along the Xi, thereby resulting in monoallelic expression of most of X-linked genes, so as to compensate for dosage inequalities with males who only have one X chromosome. In the mouse, extraembryonic tissues feature preferential silencing of the paternal X chromosome, possibly a remnant of its partially pre-inactivated state at the time of fertilization. Green boxes indicate expressed genes, gray boxes indicate silenced genes. “~X~” represents most genes on the X chromosome outside of the XIC.

The molecular underpinnings of XCI are still not fully understood, but a 500 kb stretch of DNA at Xq13 known as the X-inactivation center (XIC) is of key importance. Within this locus is a 100 kb core region containing several lncRNAs—X-inactive specific transcript (*Xist)*, *Xist’*s antisense transcript *Tsix*, X-inactivation intergenic transcription elements *(Xite),* Jpx transcript, Xist activator *(Jpx)*, and others—that play crucial roles in XCI [27]. *Xist* was one of the first identified lncRNAs, and is a ~17 kb transcript (~19 kb in humans) expressed from the future inactive X chromosome (Xi) [28]. *Tsix* is a ~40 kb transcript that is antisense to, and negatively regulates, *Xist* (see Figure 2). Furthermore, *Xite* seems to be a transcriptional enhancer of *Tsix* [27], and likewise, *Jpx* RNA appears to be required for *Xist* expression [29]. In humans, *XIST* expression is initiated as early as the eight-cell stage [74], and expression of Xist is visible in mouse embryos at the eight-cell stage [75].

*Tsix* and *Xite* are believed to function in the counting mechanism in pluripotent embryonic cells that are beginning the process of differentiation, where the two homologous X chromosomes are brought together in close proximity, specifically at the XIC [20,30,31]. This “chromosome kissing” requires *TSIX* and *XITE*, and is associated with the presence of RNA Polymerase II (RNAPII) [20,32]. Furthermore, this process is also heavily reliant on the chromatin insulator CTCF, which is known to bind at the *TSIX* and *XITE* genomic loci [33]. Molecular cross-talk across this RNA-protein “bridge” can then occur [27], where it is believed that pluripotency-related transcription factors such as OCT4, which are present on the *TSIX* promoters of both X chromosomes at the time, are thermodynamically dumped onto one of the chromosomes, which then becomes the active X chromosome (Xa), based on its continued expression of *TSIX* [31]. Thereafter, DNMT3A is recruited to the Xa and establishes *stable* silencing of *XIST* on the Xa [20,27,29].

On the homologous X chromosome (the future Xi), *Tsix* is now downregulated, which de-represses *Xist* *in cis*, although it is not known whether the *Tsix* transcript itself or the *act* of transcription is what inhibits *Xist* expression in the first place. The *Xist* transcript is capped, spliced, and polyadenylated, but escapes export [19]. The rising levels of *Xist* RNAs accumulate and are tethered *in cis* to the Xi with the help of the bivalent (DNA and RNA) associations of the transcriptional regulator YY1, which then allows the *Xist* RNA to spread and form an RNA “cloud” coating the Xi *in cis* [34]. *Xist* also recruits a chromatin modifier, the Polycomb Repressive Complex 2 (PRC2) to the Xi [29,35]. The spreading of the *Xist* cloud across the Xi begins with *Xist* first associating with ~150 strong PRC2 binding sites (CpG islands) along the chromosome, followed by association with 3000–4000 moderate-strength binding sites, thus spreading in many gradients nucleated at multiple locations on the chromosome [36]. PRC2 is believed to be initially recruited to the XIC and to the *Xist* transcripts by the action of another lncRNA called *RepA*, a 1.6 kb independently-transcribed segment of Exon 1 of *Xist* harboring conserved satellite (“A”) repeats, which are likewise also present on the 5’ end of *Xist* transcripts (see Figure 2) [20,27,32]. This interaction with the “A” satellite repeats is mediated by EZH2, a member of the PRC2 complex [37]. In addition to YY1 and *RepA*, *Xist* also interacts with the nuclear matrix-binding protein hnRNP U. hnRNP U has RNA and DNA binding domains, both of which are required for Xi formation [76]. Once settled along the Xi with the spreading *Xist* transcripts, PRC2 is responsible for marking the chromatin with H3K27 trimethylation (a repressive histone modification), which occurs in conjunction with the loss of active histone marks such as H4 acetylation, as well as H3K4 and H3K36 methylation [29,33]. In hnRNP U deficient cells, this H3K27 repressive mark is not established, leading to defects in XI formation [76,77]. After the deposition of these histone H3 marks, histone H2A is replaced with the variant *macro*-H2A [33]. Furthermore, DNA methylation occurs within the CpG islands within silenced genes on the inactivated X chromosome [99,105], which, along with the aforementioned histone modifications, are all maintained in daughter cells and serve to silence the vast majority of genes on the Xi.

Importantly, XCI is reversible in the initiation phase, but stable thereafter, when the Xi is condensed into a constitutive heterochromatic state [32]. Furthermore, *Xist* RNA is required for Xi establishment, but not strictly necessary for XCI maintenance, although it does play a role in the stability of XCI, in conjunction with PRC1 and PRC2 [30].

Interestingly, a recent report identified a novel lncRNA, far away from the XIC, that may be involved in XCI [38]. This gene, named *XACT*, is located at Xq23, about 40 Mb away (telomeric) from the XIC, in an unusually desolate area of the X chromosome, and is poorly conserved in the mouse. It produces a very large ~252 kb nuclear-retained unspliced transcript that is expressed only from the Xa, and only in pluripotent stem cells [38]. Furthermore, this lncRNA is thought to coat the Xa in a manner similar to the way *Xist* coats the Xi [38], although the mechanism that facilitates this coating has not yet been studied. It is hard to say, though, what role *XACT* may have in XCI, given the well-known instability of XCI in hESC cells. It was observed in hESC cells that *XACT* transcription was monoallelic when *XIST* was expressed, but biallelic when *XIST* was not expressed [38], suggesting a negative/reciprocal expression relationship. The authors postulated that expression of *XACT* could protect the Xa from inactivation; however, this seems incongruent with the *in vivo* notion that *XACT* would be expressed in preimplantation embryos, where *XIST* is known to be biallelically expressed [39]. In any case, this RNA and its effects on XCI will require further study.

### 2.1. Imprinted XCI

It is known that both sex chromosomes are inactivated during meiosis in males, and only partially reactivated after completion of spermatogenesis [36]. By virtue of the fact that a paternal X chromosome (X^P^) obtains its unique chromatin signature during gametogenesis, and is delivered to the ovum in a partially pre-silenced state, it can therefore be considered imprinted. The X^P^ maintains its partially-silenced state through the earliest stages of embryogenesis. Thereafter, reactivation of the X^P^ occurs in the epiblast cells at around the implantation stage, followed by random inactivation in the somatic cells of the developing embryo [27].

No germline-derived DMR has been identified at the XCI or anywhere on the X-chromosome to our knowledge, and none is documented in the comprehensive database accessible at http://www.mousebook.org/catalog.php?catalog=imprinting. This implies that imprinted XCI is most likely not dictated by an ICR, but by the overall chromatin signature of the pre-fertilization X^P^.

Although it is well established that imprinted XCI is maintained in extraembryonic tissues in the mouse as a remnant of the semi-inactivated state of the X^P^, there is evidence that this does not occur in humans [40], and some theories invoke the example of human Turner Syndrome (XO genotype) as an illustration [36]; that is, if preferential silencing of the X^P^ were maintained in extraembryonic tissues, then X^M^O embryos would not be affected, but X^P^O embryos would have limited expression of X-linked genes in those tissues, which could be problematic.

An interesting and somewhat related possibility is that nonrandom XCI (or at least some kind of differential regulation of X-linked genes, such as partial imprinting) occurs in the brain, as significant differences have been documented between X^M^O and X^P^O girls in terms of brain structure and function, as well as social adjustment [36]. If a special brain-specific XCI mechanism does exist, it is then plausible, by extension, that some of the well-documented cognitive and behavioral differences between males and females are due, in part, to differences in X-linked gene expression, as the X^P^ is usually only inherited by females.

An exception to this, however, would be some Klinefelter Syndrome cases. Although no distinctions have been identified in mental/social abilities between X^M^X^M^Y *versus* X^M^X^P^Y males (these genotypes occur at nearly equal ratios in the population [41]) to our knowledge, it has been shown that the X^P^ is associated with later-onset and slower progression of puberty [42]. However, Klinefelter patients often have skewed XCI in one direction or another in all tissues (for both genotypes) [41], and although it is not known whether this somatic XCI skewing is due to some kind of imprinting mechanism or other factor, it would nevertheless be difficult to determine whether the genotype directly produced those (or any other) differences in the subjects. More pertinent to imprinted XCI, though, it has been shown in mice that X^M^X^M^Y and X^M^X^P^Y fetuses had different extraembryonic growth properties [43], and it would be interesting to see if the differences extended to behavior patterns or cognitive abilities postnatally, although that phenomenon has not been studied.

The silencing mechanisms are thought to be mostly similar between random XCI and imprinted XCI, but more pertinent to this review is knowing what are the unique properties of *imprinted* XCI. To that end, some interesting differences between imprinted XCI and random XCI have been documented in mice. For example, it is known that the somatic Xi replicates slightly later in the cell cycle, whereas the extraembryonic (imprinted) Xi replicates a bit earlier [78]. The reason for this is not clear, although it is speculated to be related to differences in the epigenetic states between the inactive Xs [30]; that is, the imprinted Xi has less CpG methylation than the somatic Xi [44] In addition, it has been described that *Tsix* and *Xite* are not required to mark the Xa [33], and that *Xist* is not required for initiation of silencing, but is required for maintenance—parameters opposite of those in random XCI [27]. As for other special factors, experiments have shown that EED [79] (a member of PRC2) is required for imprinted XCI, but not for random XCI [33]. In addition, the DNA methyltransferase, Dnmt1, was shown to be important for stable silencing of the Xi in somatic tissues, but dispensable for placental (imprinted) XCI maintenance [44].

## 3. Kcnq1/Kcnq1ot1

**Figure 3:**
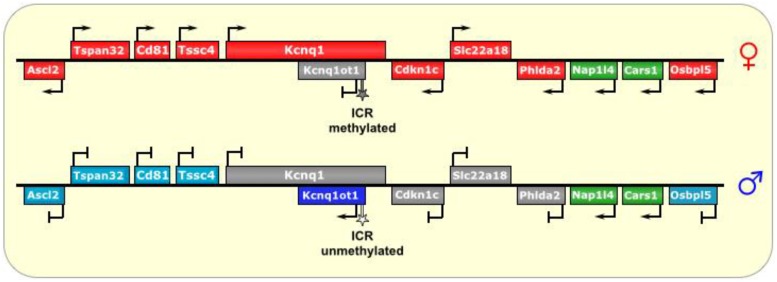
Kcnq1/Kcnq1ot1. The ICR of this imprinted locus is at the promoter of the lncRNA *Kcnq1ot1*. On the maternal allele, it is methylated, thus preventing its transcription. On the paternal allele, the ICR is unmethylated, allowing transcription of Kcnq1ot1, which is antisense to part of the *Kcnq1* gene. The lncRNA then spreads bi-directionally in a cloud-like manner throughout the locus and silences several genes *in cis.* In placental tissues, the silencing effect has an even wider reach. ICR is depicted as a star lollipop; white is unmethylated, gray is methylated. Red boxes indicate maternally expressed genes, blue boxes indicate paternally expressed genes, green boxes indicate biallelically expressed genes, gray boxes indicate silenced genes, and cyan boxes indicate genes that are (paternally) silenced in placental tissues only.

The *Kcnq1* region is a well-studied ~800 kb imprinted locus located on human chromosome 11p15 and mouse chromosome 7 [32], with several maternally-expressed protein-coding genes (including *Kcnq1* itself, coding for potassium voltage-gated channel, KQT-like subfamily, member 1), and a 60 kb paternally-expressed unspliced lncRNA *Kcnq1* opposite strand/antisense transcript 1 (*Kcnq1ot1*) that is antisense to an interior portion of *Kncq1* and serves to silence it *in cis*, as well as nearby genes (bi-directionally) on the paternal chromosome (See Figure 3) [45,46,47]. This antisense noncoding RNA initiates transcription midway through the (sense) Kcnq1 gene, from a promoter in Intron 10 [16,45]. The *Kcnq1ot1* promoter has in fact been determined to be the ICR of this imprinted region. It is methylated in oocytes, and subsequently, on the maternal allele in the embryo and extraembryonic tissues, to prevent expression of the *Kcnq1ot1* lncRNA [33,47]. Paternal transmission of an ICR deletion results in no expression of *Kcnq1ot1* and biallelic expression of the maternal genes in mice, while a maternally-inherited deletion has no effect on imprinting [16]. Likewise, early truncation of the *Kcnq1ot1* RNA results in biallelic expression of the (otherwise) maternal genes in the cluster, although some studies showed varying levels of imprinting with respect to different length truncations of the lncRNA [13,16].

Repeat elements at the 5’ end of *Kcnq1ot1* have been identified as important for the repression of the imprinted genes in the locus [30,37], and may function by targeting the genomic locus to the perinucleolar region, which is full of silencing factors [32]. Interestingly, two CTCF binding sites were identified at the *Kcnq1ot1* ICR, which were only found to be occupied on the unmethylated paternal allele [13], suggesting the need for protection against the repressive chromatin surrounding it, as nearly the entire paternal locus (except of *Kcnq1ot1*) exists in a heterochromatic state devoid of RNAPII [32,35].

In addition, here too, members of the PRC2 complex, specifically EED [33,79], as well as Ezh2 [80] and Suz12 [35,37,81,82], are associated with silencing of the genes within this imprinted cluster, and may be involved in bridging the lncRNA with the chromatin [45] and with recruiting other chromatin-altering factors, such as histone methyltransferases and histone deacetylases [49]. Indeed, the silencing of the locus is linked to the presence of the repressive histone marks H3K9me3 and H3K27me3, mediated in part by the histone methyltransferase G9A/EHMT2 [35].

In addition, the DNA methyltransferase Dnmt1 is believed to have an important role in maintaining DMR methylation at the *Kcnq1* locus in somatic tissues, but not in placental tissues, suggestive of different imprinting mechanisms at work within different tissues [33,50]. Furthermore, imprinting-mediated chromatin remodeling appears to be more tightly regulated and more closely tied to EZH2 and G9A/EHMT2 in the placenta (as opposed to somatic tissues), where it produces a wider area of imprinting (See Figure 3) with respect to somatic tissues [13,18,51], again highlighting the possibility of additional factors that augment the placental-specific imprinting of this locus [46].

In theory, the formation of dsRNA from opposing transcripts is a formal possibility, although it is thought that the silencing phenomenon by *Kcnq1ot1* is independent of the RNAi pathway [11,18,48]. *Kcnqot1* RNA is known to form a cloud (in a manner reminiscent of *Xist*, but on a smaller scale) around the locus, and was also found to occupy the promoters of the paternally silenced genes in the locus *in cis* [18]. However, other work has shown that RNAi knock down of the *Kcnq1ot1* RNA did not change the imprinting status of genes in this locus [83]. Furthermore, in a conditional knockout model that removed the promoter for *Kcnq1ot1* it was shown that the *Kcnq1ot1* RNA is critical for imprinting mediated silencing in most tissues, while in the placenta silencing of a subset of genes was maintained, despite the absence of *Kcnq1ot1* RNA [52]. Interestingly, some genes within the *Kcnq1* locus are not imprinted. These genes were found to bear active chromatin marks (H3K4me1 and H3K27ac) on their enhancers, perhaps acting as protective barriers against the repressive chromatin surrounding them, and/or against the effects of *Kcnqot1* lncRNA [52]. Together, these data indicate a complex mechanism of imprinting that include RNA-, chromatin-, and transcriptional-regulation of imprinting.

## 4. Igf2r/Airn

*Igf2r*/*Airn* is a medium-sized ~500 kb imprinted locus found on human chromosome 6q26 and mouse chromosome 17 [47] and shares many properties with the *Kcnq1* imprinted locus—where a set of maternally-expressed protein-coding genes is silenced on the paternal chromosome *in cis* by a paternally-expressed lncRNA that is antisense to one of the central maternal genes. While Igf2r is imprinted in mice, and has been shown to be maternally hypermethylated in a CpG island in humans, it is biallelically expressed in almost all tissues examined [84,85,86], with the exception that monoallelic expression has been noted in rare fetal samples [87,88] and samples from Wilm’s tumors [89].

**Figure 4:**
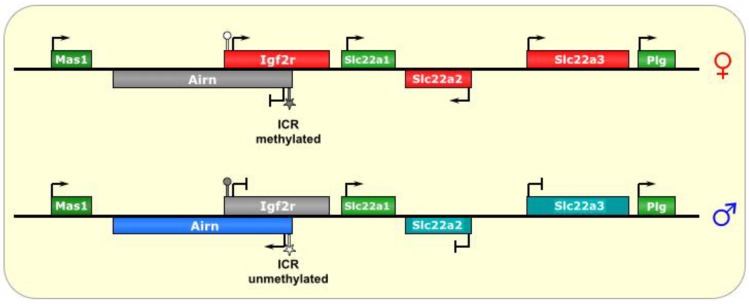
Igf2r/Airn. The ICR of this imprinted locus is at the promoter of the lncRNA Airn. On the maternal allele, it is methylated, and, thus, silenced. On the paternal allele, the ICR is unmethylated, allowing transcription of *Airn*, which is antisense to *Igf2r* and silences it *in cis* via transcriptional interference. In the placenta, the silencing effect has an even wider reach, although it is not known what tissue-specific factors regulate this process. ICR is depicted as a star lollipop, and other DMRs as circle lollipops; white is unmethylated, gray is methylated. Red boxes indicate maternally expressed genes, blue boxes indicate paternally expressed genes, green boxes indicate biallelically expressed genes, gray boxes indicate silenced genes, and cyan boxes indicate genes that are (paternally) silenced in placental tissues only.

*Igf2r* encodes the Insulin-like Growth Factor 2 Receptor, and is expressed from the maternal chromosome [37]. Two other maternal genes in the cluster are *Slc22A2* and *Slc22A3*. The paternal lncRNA that regulates the imprinting in this cluster is called *Air* (also known as *Airn*, which stands for Antisense Igf2r RNA Noncoding) [53]. The *Airn* transcript is ~118 kb and mostly (95%) unspliced [11]. It is antisense to part of *IGF2R* (but not all of it), and does not extend into the other genes in the locus, as they are all upstream of its promoter (see Figure 4); however, *Airn* does overlap with the *Igf2r* promoter [54]. An *Airn* homolog was only recently identified in human (*AIR*), and it is not yet clear what its role is, as *IGF2R*, *SLC22A2*, and *SLC22A3* are biallelically expressed in humans [90].

The ICR for this imprinted cluster is located at the promoter of *Airn*, within Intron 2 of the *Igf2r* gene (on the opposing DNA strand) [27], and repeat sequences found at this DMR have been shown to be important for its methylation in the oocyte [47]. Interestingly, silencing of *Igf2r* does not require DNA methylation or histone modification [54]. In fact, examination of *Igf2r* chromatin did not find broad repressive histone marks, but rather, a small array at its promoter [30,53].

In addition, truncations that prematurely terminate *Airn* before it reaches the *Igf2r* promoter result in de-repression of the paternal *Igf2r* allele [53]. Furthermore, repositioning of the *Airn* promoter (or an exogenous promoter) very close to the *Igf2r* promoter, such that it still transcribes through it (in the antisense direction) but without producing the *Airn* transcript, resulted in normal imprinting of *Igf2r* [54]. Together, the model of imprinting in this locus, at least for *Igf2r*, appears to be one of transcriptional interference by blocking RNAPII from the promoter of *Igf2r*, even in the face of a generally less restrictive chromatin state, rather than a model of RNA-mediated silencing by recruitment of Polycomb complexes or other chromatin remodeling factors, although it is very difficult to demonstrate this directly [54].

As for the other genes of the locus, a wider range of silencing is known to occur in the *Igf2r* locus in extraembryonic tissues with respect to ubiquitous silencing (similar to the *KCNQ1* locus), where *Igf2r* is imprinted in all tissues, while *Slc22A2* and *Slc22A3* are only imprinted in extraembryonic lineages [3,55]. Again, this suggests the presence of alternative (or additional) factors that function in placental imprinting [18]. Indeed, the histone methyltransferase G9A/Ehmt2 has been implicated in the placental-specific imprinting of this locus [54], a phenomenon dependent on the accumulation of *Airn* RNA at the promoter of the silenced gene [47,56]. Another interesting similarity to *Kcn11* imprinting is that two of the genes in the *Igf2r/Airn* locus (*Slc22A1* and *Mas1*) actually escape imprinting, via unknown mechanisms [13,33].

## 5. H19/Igf2

The ~80 kb *H19/IGF2* locus is one of the smallest imprinted clusters [11] and includes the maternally-expressed noncoding gene *H19*, and the paternally-expressed protein-coding IGF2 gene located about 90 kb away [1]. These two reciprocally regulated genes share an enhancer, located downstream of *H19*, distal to *IGF2* [12]. The ICR is located between the two main genes, about 2–4 kb upstream of the *H19* transcriptional starts site (TSS) [1], and contains four binding sites for the chromatin insulator CTCF [12]. This imprinted cluster is found just upstream (~100 kb) of the *KCNQ1* locus at chromosome 11p15, but despite their proximity, *H19*/*IGF2* and *KCNQ1*/*KCNQ1OT1* are completely separate domains, and are independently regulated [16].

**Figure 5:**
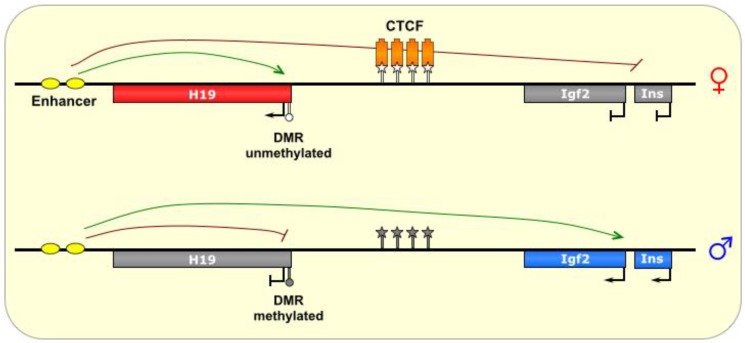
H19/Igf2. The ICR of this imprinted locus is intergenic, located between the *Igf2* gene and the noncoding *H19* gene, which share an enhancer (*yellow ovals*). On the maternal allele, the ICR is bound by the insulator protein CTCF (*orange boxes*), which prevents methylation of the ICR. The formation of chromatin loops defines a transcriptionally favorable domain that allows *H19* access to the enhancer, and prevents transcription of the *Igf2* and *INS* genes. On the paternal allele, the ICR is methylated, thereby allowing transcription of *Igf2* and *Ins*, and silencing *H19*. ICR is depicted as a star lollipop, and other DMRs as circle lollipops; white is unmethylated, gray is methylated. Red boxes indicate maternally expressed genes, blue boxes indicate paternally expressed genes, and gray boxes indicate silenced genes.

On the maternal chromosome, CTCF binds at the ICR and creates an insulated domain, which prevents *IGF2* from accessing the shared enhancer and results in its silencing (see Figure 5) [13]. The mechanism of CTCF’s insulation effect is thought to be mediated by the formation of chromosomal loops that define and protect a domain (euchromatic *versus* heterochromatic) by preventing the spread of chromatin marks from nearby regions [57]. The precise orientation of the CTCF loops at the *H19*/*Igf2* ICR is unknown, although hypotheses abound regarding the possible physical interactions that play out with CTCF at this ICR and result in the functional inhibition of *Igf2* transcription [13]. Additional factors, including cohesins, have also been shown to be important in CTCF function at this locus, although their role in the process is not well-known [12,13].

However, in the paternal germline, DNA methylation marks are added onto the ICR by the *de novo* DNA methyltransferase DNMT3L [12], and then spread to the *H19* promoter as well [14], which precludes CTCF binding and presumably alters the formation of chromosomal loops, thereby allowing *Igf2* to access the enhancer and be transcribed, at the cost of *H19* activation [1,13]. These methylation marks are thought to be maintained in part by MBD3, of the NuRD complex [13]. Interestingly, the ICR alone is not sufficient for DNA methylation to occur on the paternal allele, and requires additional (as of yet unidentified) sequences from the locus to establish methylation [12].

It is thought that CTCF acts to prevent DNA methylation at the *H19* ICR, as artificial prevention of CTCF binding (via binding site mutation or CTCF knockdown) has been shown to cause aberrant methylation of the ICR in the maternal lineage, thereby adopting a paternal epigenetic conformation [14]. Nevertheless, the paternal methylation of the allele is not necessarily its default state, as other factors may regulate this process specifically in the male germline [14], such as the CTCF-like transcriptional repressor BORIS (also known as CTCFL) [13]. Interestingly, the conserved presence of CTCF binding sites at this locus suggest that *H19/IGF2* is evolutionarily the oldest imprinted cluster [13].

*H19* produces a spliced ~2.3 kb noncoding transcript [58] that is believed to function as an oncogene [25]. However, the *H19* transcript is also a precursor for the microRNA *MIR675*, which has been shown to inhibit placental growth [58]. The reciprocally expressed *IGF2* gene codes for a fetal growth factor [13]. Interestingly, *H19* is only expressed in the pons and globus pallidus in the adult human brain [91], and consequently, *IGF2* is biallelically expressed in other regions of the brains, suggesting tissue-specific factors that differentially regulate the expression of these genes in the brain [32]. However, in fetal brain, *H19* is widely expressed [91], and thus *IGF2* is monoallelically expressed in these tissues.

The *H19*/*Igf2* locus also produces several antisense transcripts. Both *Igf2* and *H19* have antisense transcripts, each, surprisingly, having the same allele-specific pattern of transcription as its sense transcript [58]. Interestingly, the *H19* area produces two different types of antisense transcripts with seemingly opposing functions—a nuclear 120 kb noncoding isoform called *91H* that is associated with tumorigenesis, and a 6 kb coding form called *HOTS*, whose protein product has been identified mostly in the nuclei of fetal tissues and is believed to function as a tumor-suppressor [58]. Although *91H* is not believed to regulate *H19* expression, it does seem to have a surprising *positive* effect on *Igf2* expression [58].

The *H19*/*Igf2* region is thought to be critically important in embryonic development [6,7,8,9,10], and may also have a role in regulating lifespan [59]. Disruptions of this locus by mutation or aberrant imprinting are linked to Beckwith-Wiedemann Syndrome and/or Silver-Russell Syndrome [1,25], as well as several cancers [25,59].

## 6. DLK1-DIO3

**Figure 6:**
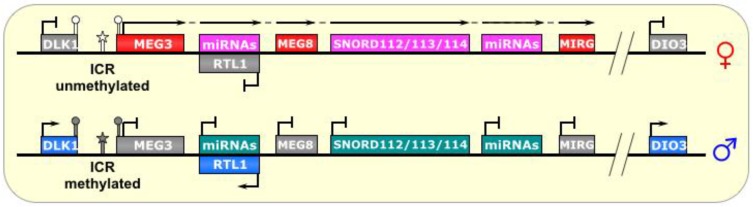
Dlk1-Dio3/Meg3. The ICR of this imprinted locus is just upstream of the lncRNA *MEG3*. On the paternal allele, it is methylated, thus, preventing its transcription. On the maternal allele, the ICR is unmethylated, allowing transcription of *MEG3* and other genes in a polycistronic fashion, which includes a segment that is antisense to *RTL1* and silences it *in cis*. This long transcript also produces several snoRNAs and miRNAs. ICR is depicted as a star lollipop, and other DMRs as circle lollipops; white is unmethylated, gray is methylated. Red boxes indicate maternally expressed genes, pink indicates maternally expressed small RNA clusters, blue boxes indicate paternally expressed genes, gray boxes indicate silenced genes, and cyan boxes indicate silenced small RNA clusters. Dotted-tail arrow indicates contiguous transcription.

Human chromosome 14q32 and chromosome 12 in mouse is another region bearing a large imprinted cluster: *DLK1-DIO3* is a ~1 Mb region flanked by the paternally-expressed genes *DLK1* and *DIO3*, with an interior containing maternally-expressed noncoding RNAs, including snoRNAs, miRNAs, and most notably, the lncRNA *MEG3* (also known as *Gtl2*) [60]. This highly-conserved region is expressed prenatally in a variety of tissues, but postnatally limited primarily to the brain [61]. As depicted in Figure 6, the maternal allele also transcribes a segment antisense to the *RTL1* gene containing two miRNAs complementary to *RTL1*, which are believed to downregulate the maternal *RTL1* transcript [61,62]. This anti-*RTL1* RNA may also be polycistronic/contiguous with the transcripts that host multiple snoRNA clusters (the SNORD112s, SNORD113s, and SNORD114s), as well as several miRNAs, and the lncRNA *MEG8* (also known as *Rian* in mouse), starting with *MEG3* and ending with *MIRG* [62].

Three paternally-methylated DMRs have been identified upstream of *Meg3* and have varying degrees of influence on the imprinting status of this region [60]. The interior DMR, called the IG-egDMR, is the ICR of this region. Interestingly, although it is methylated on the paternal chromosome, paternally-inherited deletion showed no effect in mice. Conversely, maternal transmission de-regulated the imprinting of the entire region, as revealed by biallelic *Dlk1* expression and biallelic *Meg3* silencing [63]. This indicates that the IG-DMR is not involved in silencing the paternal *Meg3* transcript, but is necessary (in the unmethylated state, perhaps acting as an activator) for expression of the maternal *Meg3* and silencing the maternal *Dlk1* gene [63]. Like *Dlk1*, it is known that *Dio3* also has a reciprocal expression pattern as (and may be regulated by) *Meg3*, although it is much further away, and the mechanism by which this silencing occurs is not known.

*Meg3* knockdown also revealed a two-fold increase in *Dlk1* expression, suggesting reactivation of the maternally imprinted (silenced) allele [64]. Several possibilities exist as to the mechanisms of this interesting regulation. First, the *Meg3* lncRNA could recruit positive chromatin factors as well as DNA demethylases, or even transcription factors, to its own gene locus. Conversely, it could titrate negative chromatin regulators away from the IG-DMR and perhaps target them to *Dlk1* instead. Indeed, *Meg3* knockdown resulted in a decrease in EZH2 (a subunit of the PRC2 complex) recruitment to the *Dlk1* promoter, and a corresponding decrease in the PRC2-mediated chromatin mark H3K27me3, consistent with *Dlk1* upregulation, which was similarly seen upon *EZH2* knockdown [64]. In addition, an unexplored possibility is that *Meg3* could act as a competing endogenous RNA (ceRNA) against miRNAs that regulate either of the aforementioned processes.

Interestingly, evolutionary studies on this cluster have indicated that its imprinted status coincided with the appearance of ncRNAs in the locus, notably a lncRNA, as the marsupial *DLK1* region is neither imprinted nor contains a lncRNA [61]. In addition, deletion or aberrant imprinting of this region has been linked to tumorigenesis in mammals [25].

## 7. SNURF-SNRPN

**Figure 7:**
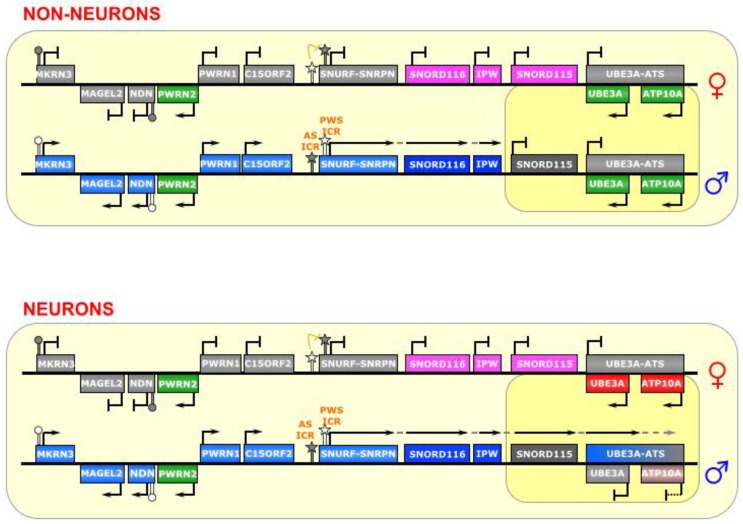
SNURF-SNRPN/UBE3A. This region contains a bipartite imprinting center consisting of two reciprocally regulated ICRs—the AS-ICR (proximal) and the PWS-ICR (distal). The latter is epistatic to the former; that is, on the maternal allele, the AS-ICR is unmethylated, and allows for the methylation of the PWS-ICR, which falls within the promoter of the lncRNA *SNURF-SNRPN* (as well as several other genes upstream of it), thus silencing those genes. On the paternal allele, the AS-ICR is methylated, and the PWS-ICR is unmethylated, allowing the transcription of *SNURF-SNRPN*. In most cell types, the long *SNURF-SNRPN* transcript stops at the *IPW* gene, and the *UBE3A* gene is biallelically expressed. However, in neurons, the *SNURF-SNRPN* transcript extends past IPW, and through *UBE3A* (and possibly *ATP10A*) in the antisense direction, thereby silencing them. The long polycistronic transcript also produces many snoRNAs. ICRs are depicted as star lollipops, and other DMRs as circle lollipops; white is unmethylated, gray is methylated. Red boxes indicate maternally expressed genes, blue boxes indicate paternally expressed genes, green boxes indicate biallelically expressed genes, gray boxes indicate silenced genes, pink/gray boxes indicate genes that are partially (maternally) silenced, and small RNA genes that are processed from the host transcript are noted. Dotted-tail arrow indicates contiguous transcription.

The SNURF-SNRPN region is the largest known imprinted cluster, comprising a >2 Mb area on chromosome 15q11-13 (See Figure 7), and is associated with the neurodevelopmental disorders Angelman Syndrome (AS) and Prader-Willi Syndrome (PWS) [16]. Although many of its imprinting rules are known, there is still much to understand about the complex gene regulation that takes place there. The region contains several protein coding genes, including *SNURF-SNRPN* and *UBE3A*, as well as numerous noncoding genes. Some of these noncoding genes are transcribed from a long polycistronic RNA that starts at the *SNURF-SNRPN* promoter; thus, the RNA is bifunctional.

This region is controlled by elements within the *SNURF-SNRPN* promoter, which is composed of a bidirectional activator responsible for a number of genes in this locus [65], making it the epicenter of regulation in this imprinted cluster. It is a bipartite imprinting control center; that is, two (reciprocal) ICRs are at play here—the 4.3 kb PWS-IC (which includes Exon 1 and the promoter of the *SNURF-SNRPN* gene), and the 0.88 kb AS-IC (located 35 kb upstream) [70]. It is believed that the latter specifically and unidirectionally inhibits the former, although the regulatory mechanism is not fully known [65,71]. Indeed, experiments have shown that the AS-IC is essential for the methylation of the PWS-IC [72], which seems to be the result of transcriptional activity through the PWS-IC in oocytes [92]. Interestingly, a relatively small deletion in the mouse *Snurf-Snrpn* promoter prevents expression of *Snrpn* transcript, if inherited paternally, but leaves imprinting of other genes in the region (Ndn, Magel2, and Mkrn3) intact [93]. The *SNURF-SNRPN* lncRNA covers an area of 170–600 kb area, depending on the tissue, and is host to dozens of small nucleolar RNAs (snoRNAs) [66], as well as other unusual ncRNAs, including snoRNA-bound lncRNAs (sno-lncRNAs) [67] and snoRNA-derived short/processed RNAs (sdRNAs or psnoRNAs) [68,69]. As paternal imprinting of *SNURF-SNRPN* dictates that it is monoallelically expressed in all tissues, the other RNAs derived from this large transcription unit (i.e., *IPW*, *SNORD116*, and other snoRNAs) are also monoallelically expressed [67].

Typically, in the female germline, the AS-IC is active and later able to shut down the PWS-IC (via CpG-methylation and H3K9-trimethylation), which happens to also include the *SNURF-SNRPN* promoter, and thus inhibits transcription of the maternal allele and renders *SNURF-SNRPN* expression as paternal-only. Conversely, the AS-IC is inactive in the male germline, which allows the PWS-IC DMR to stay unmethylated (although it is still bears the active marks H3K4 di- and tri-methylation), leaving it primed for transcription [22,72].

The PWS-IC acquires the DNA methylation required for imprinting during gametogenesis both in human [94] and in mouse [95,96]. Similarly, it is clear that the inhibitory AS-IC bears the primary imprinting mark from the germline, and later confers a reciprocal mark onto the permissive PWS-IC, which then becomes the major post-fertilization imprinting regulator [65], as the AS-IC loses its differentially methylated status later in development and is, thenceforth, dispensable for maintenance of imprinting [72]. Furthermore, double deletions of both the AS-IC and the PWS-IC, passed through either the maternal or paternal lineage, reveal a phenotype consistent with paternal genes not being properly expressed, suggesting a bi-maternal chromosomal configuration, which means that the PWS-IC is epistatic to the AS-IC [70].

In neurons, an unknown mechanism changes the transcriptional regulation of the *SNURF-SNRPN* lncRNA, allowing it to continue transcribing over a total of 500 kb, past *IPW* and the *SNORD115* cluster, and through the *UBE3A* gene in the antisense direction (*UBE3A-ATS*) (see Figure 7) [66]. A gene adjacent to *UBE3A*, *ATP10A*, may also be affected by this imprinting, as it is imprinted in some humans [97], but has been shown to not be imprinted in mouse [98]. This antisense transcription silences the paternal alleles of *UBE3A* and, in perhaps is some cases, *ATP10A*—causing maternal-only expression in neurons. It is unclear, however, whether it is the act of transcribing or the antisense lncRNA itself that governs this silencing mechanism on the paternal allele in these cells.

In keeping with the theme of this review, we will not discuss in detail the short RNAs (over 75 snoRNAs, including the *SNORD115* and *SNORD116* clusters) derived from this imprinted locus, but we would like to note some interesting lncRNAs recently identified in this region. Increased attention has been paid to the region containing the *SNORD116* cluster, as a deletion encompassing these snoRNAs has been found to be the minimal deletion sufficient to cause PWS [100,101,102]. Three recent reports have uncovered novel RNAs derived from this region that may possibly play a role in the etiology of diseases associated with this locus. One report identifies a role for the *Snord116* host gene (*116HG*) RNA as a chromatin binding lncRNA cloud that interacts with genes important for metabolic regulation [103]. These initial findings were extended to a *Snord116* deletion mouse model of PWS, and overlap between significantly altered transcripts and *116HG* interacting genes in the cortex indicates that *116HG* has a role in the dysregulated metabolic phenotype observed in the mouse [103].

A second study from the same group focused on the local interactions between the *Ube3a-ATS* transcript and the *Snord116* region. It reported that DNA:RNA hybrids called R-loops are formed in the *Snord116* region, as a result of its high GC content [104]. In cells without *Snord116* deletions, these R-loops create a balance between RNA PolII stalling and transcriptional elongation, which results in *Ube3a-ATS* and silencing of *Ube3A*. This balance can be upset, however, either by deletion of the repetitive *Snord116* region leading to increased transcriptional activity of *Ube3a-ATS*, or addition of the topoisomerase inhibitor topotecan leading to increased R-loop formation and decreased *Ube3a-ATS* [104]*.*

A third recent report revealed a novel class of lncRNAs, called sno-lncRNAs, which arise from the unusual presence of two snoRNAs within the same intron [67]. These RNAs were first identified as arising from the *SNORD116* cluster, although they have been found to arise from other genomic regions. The biogenesis of these unique lncRNAs is believed to occur via the same mechanisms as canonical splicing-dependent snoRNA processing; that is, after intron excision, exonucleolytic cleavage of the debranched lariat proceeds until it is obstructed at the site where the set of proteins comprising the snoRNP complex forms, (in this case, bilaterally), with the intervening sequence undigested, leaving highly stable 1–3 kb lncRNA molecules with a snoRNA at each end, and no 5’ cap or polyA tail. Additional characterizations of the five sno-lncRNAs arising from the *SNORD116* cluster (in the 15q11-13 locus) revealed that they were rich in binding sites for members of the FOX family of splicing factors, and knockdown of these sno-lncRNAs produced specific splicing changes, so they may function as protein sinks to regulate gene expression [67]. More pertinent for this review, though, is that these sno-lncRNAs were found to remain localized to their site of transcription, which lead us to hypothesize that they could also potentially alter local chromatin dynamics by recruiting other enzymes, such as remodeling factors or methyltransferases, as do other lncRNAs [67].

## 8. Miscellaneous and Summary

The first long noncoding RNAs discovered were those involved in genomic imprinting and X-inactivation, but our knowledge of both lncRNA biology and the mechanisms of imprinting are still in its infancy. In theory, lncRNAs are an ideal candidate for the seemingly impossible and highly complex *in cis* regulation that takes place at imprinted loci, such as directing chromatin modifiers to specific locations, as they remain tethered to the site of transcription for a significant amount of time, and provide unmatched temporal and spatial specificity [31,64].

One of the greatest mysteries of imprinting is how the ICRs retain their methylation status in the face of global demethylation events after fertilization. To date, no universal factor has been identified, but ZFP57 has been shown to be important for maintenance of ICR methylation at *XIST*, *SNURF-SNRPN*, and *DLK1*, as well as non-clustered imprinted genes [15], while MBD3 maintains ICR methylation specifically at the *H19* locus [13]. In addition, STELLA (DPPA3) has been shown to have a more global protective role on methylated ICRs in the early embryo [13]. Another common thread in many imprinted clusters is PRC2, which has a role is in establishing a higher-order repressive chromatin landscape with the help of the respective signature lncRNA [13].

In many cases, it is difficult to determine whether RNA duplexes occur between the characteristic sense-antisense transcription that occurs in imprinted clusters. Although the RNAi pathway is often ruled out immediately, due to its predominantly cytoplasmic realm of function [18], to our knowledge it has not been determined whether the *nuclear* dsRNA pathway may be at work; that is, whether these RNAs are edited by the ADAR family of enzymes [73].

As mentioned above, some of these imprinted clusters not only house small RNAs, but their lncRNAs are also the precursors for those small RNAs, including snoRNAs (*SNURF-SNRPN*) [66], miRNAs (*H19*) [16,58], or both (*MEG3*) [14], which adds an additional layer of complexity to the regulation of these loci, unfortunately beyond the scope of this review.

In summary, genomic imprinting involves allele-specific epigenetic regulation of gene expression, based on parent-of-origin. Imprints are established during gametogenesis in a sex-specific manner, and are characterized primarily by DNA methylation on special DMRs called ICRs. After fertilization, these marks resist the genome-wide waves of methylation erasure, and are later complemented and strengthened by further epigenetic changes via chromatin remodeling factors and other *trans*-acting enzymes. Imprinted genes are often found in large clusters, and their expression is regulated by the transcriptional activity of an lncRNA *in cis.* Some imprinted genes also bear tissue-specific and/or temporally-regulated imprinted gene expression, particularly in extraembryonic (prenatal) and neuronal (postnatal) tissues. Despite this breadth of knowledge on imprinting, there are still many aspects that are poorly understood.
